# Spatiotemporal characteristics and the epidemiology of tuberculosis in China from 2004 to 2017 by the nationwide surveillance system

**DOI:** 10.1186/s12889-020-09331-y

**Published:** 2020-08-26

**Authors:** Zhongbao Zuo, Miaochan Wang, Huaizhong Cui, Ying Wang, Jing Wu, Jianjiang Qi, Kenv Pan, Dongming Sui, Pengtao Liu, Aifang Xu

**Affiliations:** 1 Department of Clinical Laboratory, Hangzhou Xixi Hospital, 2 Hengbu Road, Xihu District, Hangzhou, 310023 Zhejiang Province China; 2 Department of General Courses, Weifang Medical University, Weifang, 261053 Shandong Province China

**Keywords:** Tuberculosis, Spatial-temporal, Epidemiology, Multivariate time series model

## Abstract

**Background:**

China has always been one of the countries with the most serious Tuberculosis epidemic in the world. Our study was to observe the Spatial-temporal characteristics and the epidemiology of Tuberculosis in China from 2004 to 2017 with Joinpoint regression analysis, Seasonal Autoregressive integrated moving average (SARIMA) model, geographic cluster, and multivariate time series model.

**Methods:**

The data of TB from January 2004 to December 2017 were obtained from the notifiable infectious disease reporting system supplied by the Chinese Center for Disease Control and Prevention. The incidence trend of TB was observed by the Joinpoint regression analysis. The Seasonal autoregressive integrated moving average (SARIMA) model was used to predict the monthly incidence. Geographic clusters was employed to analyze the spatial autocorrelation. The relative importance component of TB was detected by the multivariate time series model.

**Results:**

We included 13,991,850 TB cases from January 2004 to December 2017, with a yearly average morbidity of 999,417 cases. The final selected model was the 0 Joinpoint model (*P* = 0.0001) with an annual average percent change (AAPC) of − 3.3 (95% CI: − 4.3 to − 2.2, *P* < 0.001). A seasonality was observed across the 14 years, and the seasonal peaks were in January and March every year. The best SARIMA model was (0, 1, 1) X (0, 1, 1)_12_ which can be written as (1-B) (1-B^12^) X_t_ = (1–0.42349B) (1–0.43338B^12^) ε_t_, with a minimum AIC (880.5) and SBC (886.4). The predicted value and the original incidence data of 2017 were well matched. The MSE, RMSE, MAE, and MAPE of the modelling performance were 201.76, 14.2, 8.4 and 0.06, respectively. The provinces with a high incidence were located in the northwest (Xinjiang, Tibet) and south (Guangxi, Guizhou, Hainan) of China. The hotspot of TB transmission was mainly located at southern region of China from 2004 to 2008, including Hainan, Guangxi, Guizhou, and Chongqing, which disappeared in the later years. The autoregressive component had a leading role in the incidence of TB which accounted for 81.5–84.5% of the patients on average. The endemic component was about twice as large in the western provinces as the average while the spatial-temporal component was less important there. Most of the high incidences (> 70 cases per 100,000) were influenced by the autoregressive component for the past 14 years.

**Conclusion:**

In a word, China still has a high TB incidence. However, the incidence rate of TB was significantly decreasing from 2004 to 2017 in China. Seasonal peaks were in January and March every year. Obvious geographical clusters were observed in Tibet and Xinjiang Province. The relative importance component of TB driving transmission was distinguished from the multivariate time series model. For every provinces over the past 14 years, the autoregressive component played a leading role in the incidence of TB which need us to enhance the early protective implementation.

## Background

Tuberculosis (TB) continues to challenge the international community. It is estimated that there are about 1.7 billion people with potential TB infection, accounting for 23% of the world’s population, are at risk of developing TB disease during their lifetime [1]. Moreover, the global burden was estimated by the World Health Organization (WHO) at 10.0 million incident cases in 2017. It is also one of the top 10 causes of death which caused an estimated 1.6 million deaths in 2017, and has killed more people than other infectious diseases in the past few decades [1, 2].

China has always been one of the countries with the most serious Tuberculosis epidemic in the world [3–6]. There were 866,000 patients with infection of TB in China, 2018 [7]. Due to the continuous attention to public health and increasing investment in resources, China’s Tuberculosis epidemic has significantly improved in recent years. However, due to the large number of people infected with TB, the epidemic situation of Tuberculosis is still not optimistic, so further long-term research on the incidence of it in China is needed.

Currently, China has conducted five national epidemiological investigations to find the epidemiological characteristics of Tuberculosis. However, the spatiotemporal distributions of Tuberculosis cannot be evaluated continuously, and the survey was unable to measure other important indicators of the severity of the epidemic. The mathematical models may help us better understand the epidemiological characteristics of Tuberculosis. Some of the studies mainly focused on the seasonality impact on the transmission of Tuberculosis [5, 8, 9], while others focused on the spatial distributions [10, 11]. There is no model that assesses the spatiotemporal characteristics and the epidemiology of Tuberculosis among the whole population in China over 14 years.

The aim of this study was to observe the Spatial-temporal characteristics and the epidemiology of Tuberculosis in China from 2004 to 2017. The incidence trend of the TB was observed by the Joinpoint regression analysis. The Seasonal autoregressive integrated moving average (SARIMA) model was used to predict the monthly incidence. Geographic clusters was employed to analyze the spatial autocorrelation. The relative importance component of TB was detected by the multivariate time series model. These models additively divided TB risks into spatiotemporal, autoregressive, and endemic components.

## Methods

### The data collection

Tuberculosis incidence data were extracted from the Chinese Center for Disease Control and Prevention (http://www.phsciencedata.cn/Share/edtShareNew.jsp?id=39208) in 31 provinces of China from 2004 to 2017. The data were aggregated to 168 monthly counts across the 14 years. Population data came from the website of the statistical yearbook of the National Bureau of Statistics (http://www.stats.gov.cn/tjsj/ndsj/). The population size was easy to find in the website, and it represented the average population each year.

### Joinpoint regression

From 2004 to 2017, the continuous change of the TB incidence trend was analyzed using Joinpoint software. The grid search method was applied to find significant trends, and multiple permutation tests were applied to detect the Joinpoint points for each trend [12–14]. The overall time trend was calculated by the annual average rate of change (AAPC). If the final model was 0 Joinpoint model, the average percent change (APC) was considered equal to AAPC. We used the Joinpoint regression model to find the long-term trend of the TB incidence.

### Time-series estimation

The SARIMA model was used to predict the future trends in many disease incidences [13, 15–17]. In our study, A SARIMA model was applied to predict the incidence of TB epidemics in China. The SARIMA model can be written as the form of (p, d, q) (P, D, Q) [s], which P, D, and Q indicate seasonal SAR terms, seasonal differencing, and seasonal SMA terms, respectively; p, d, and q indicate non-seasonal AR terms, non-seasonal differencing, and non-seasonal MA, respectively; s indicated the seasonal period (s = 12 in our study).

The construction of the SARIMA model can be divided into the following steps. First, an augmented Dickey-Fuller (ADF) test was performed to test the stationary status of time series. Second, model parameters (p, d, q, P, D, and Q) were determined by autocorrelation function (ACF) plot, partial autocorrelation function (PACF) plot, and inverse autocorrelation function (IACF) plot. An alternative SARIMA model was constructed by transforming the parameters of model. Lastly, the Akaike information criterion (AIC) and Schwartz Bayesian Criterion (SBC) were used to determine the fitness of different SARIMA models. An optimal model was considered to have the lowest AIC and SBC values, and the residuals of the final model were tested by the Box-Ljung test to know whether they were time independent. The mean square error (MSE), mean absolute percentage error (MAPE), mean absolute error (MAE), and root mean square error (RMSE) were used to see the predictive validity of the models. We use year 2004–2016 to construct the SARIMA model, and year 2017 to testify the forecast of the model. The SARIMA model is used to forecast the short-term incidence of TB to testify the accuracy of model. We also decompose the monthly data into the overall trend, seasonal trend, and random noise with a goal to identify the truly long-term trend.

### Spatial autocorrelation analysis

Spatial analysis was used to identify the clustering regions and observe geographic variation [18, 19]. Global Moran’s I of reported TB cases was computed to detect the spatial clustering pattern. A Moran’s I value is between − 1 and 1, whereas the value near 1 means positive spatial autocorrelation, the value near − 1 means negative spatial autocorrelation, and 0 means random distribution. Local Moran’s Index was calculated and a hotspot analysis was performed to determine the location of clusters. Local Moran’s Index was applied to determine the spatial autocorrelation, which detects some spatial clusters with similar adjacent features and exception values. When the incidences rate had similar low values or high values, these areas were deemed as having positive autocorrelation (low-low or high-high autocorrelation). If not, they were defined as having a negative autocorrelation (low-high or high-low autocorrelation) [10].

### The multivariate time series model

A multivariate time-series model for disease counts Y_i,t_ during periods t = 1, T from units i = 1, I was first established by Held et al [20] and was extended and applied in some papers [21–23]. The Y_i,t_ denoted the number of TB cases which were considered to be a negative binomial distribution Yit|Y_i,t-1_ ~ NegBin (u_it_, ψ), with an additively decomposed mean:

$$ {\mathrm{u}}_{\mathrm{i}\mathrm{t}}={\upsilon}_{\mathrm{i}\mathrm{t}}{e}_{\mathrm{i}\mathrm{t}}+{\lambda}_{\mathrm{i}\mathrm{t}}\;{\mathrm{Y}}_{\mathrm{i},\mathrm{t}\hbox{-} 1}+{\upphi}_{\mathrm{i}\mathrm{t}}\sum \limits_{j\ne i}{w}_{ji}{Y}_{j,t=1}, $$

Where ψ is an over-dispersed parameter that the conditional variance of Y_i,t_ is ${\mu}_{it}\left(1+\psi {u}_{it}\right)$. υ_it_ℯ_it_ is the endemic component, and the autoregressive component λ_it_Y_i,t-1_ reflects the patient numbers at previous time. The spatiotemporal component ϕ_it_ ∑j ≠ *iwjiYj*, *t* − 1 reflects the transmission among different units. Each parameter υ_it,_ λ_it_, and ϕ_it_ follow the form of log-linear:

$$ \log \left({\upsilon}_{\mathrm{i}\mathrm{t}}\right)={a}^{\left(\mathrm{v}\right)}+{{\mathrm{b}}_{\mathrm{i}}}^{\left(\mathrm{v}\right)}+\sum \limits_{s=1}^S\left\{\gamma \sin \left({w}_st\right)+\delta \cos \left({w}_st\right)\right\}, $$

$$ \log \left({\lambda}_{\mathrm{i}}\right)={\alpha}^{\left(\lambda \right)}+{{\mathrm{b}}_{\mathrm{i}}}^{\left(\lambda \right)}, $$

$$ \log \left({\upphi}_{\mathrm{i}}\right)={\alpha}^{\left(\upphi \right)}+{{\mathrm{b}}_{\mathrm{i}}}^{\left(\upphi \right)}, $$

Where α^(v)^, α^(λ)^ and α^(ϕ)^ are intercepts and b_i_^(v)^, b_i_^(λ)^ and b_i_^(ϕ)^ are random effects accounting for heterogeneity among different regions. The endemic υ_it_ contains a sinusoidal frequency wave (w_s_ = 2π/12 for monthly data), and S is the seasonal parameters. The population fraction e_i_ can be used as a multiplicative offset for the regional specific measure for the incidence of infectious disease.

The weights ω_ji_ describe the transmission from district j to district I. Considering that most regions are very large, higher-order neighbourhood are not that relevant as we only constructed our model with first-order neighbourhood. The score rule of the Dawid-Sebastiani score (“dss”) was applied to identify the optimal model with random effects. The optimal model corresponds to lower scores with better predictions [22, 24]. All the multivariate time analysis used the R package Surveillance.

### Statistical analysis

The incidence trend of TB from 2004 to 2017 was observed by the Joinpoint software (version 4.7.0.0). The Seasonal autoregressive integrated moving average (SARIMA) model was used to predict the monthly incidence of TB by SAS9.4 (SAS Institute Inc., Cary, NC). Geographic clusters was employed to analyze the spatial autocorrelation with ArcGIS software (version 10.2, ESRI Inc.; Redlands, CA, USA). The relative importance component of TB was detected by the multivariate time series model with R software (version 3.6.0, package = surveillance). *P* value < 0.05 was considered as statistically significant for all the tests.

## Results

### Time trends, seasonal characteristics of the TB incidence

**Fig. 1:**
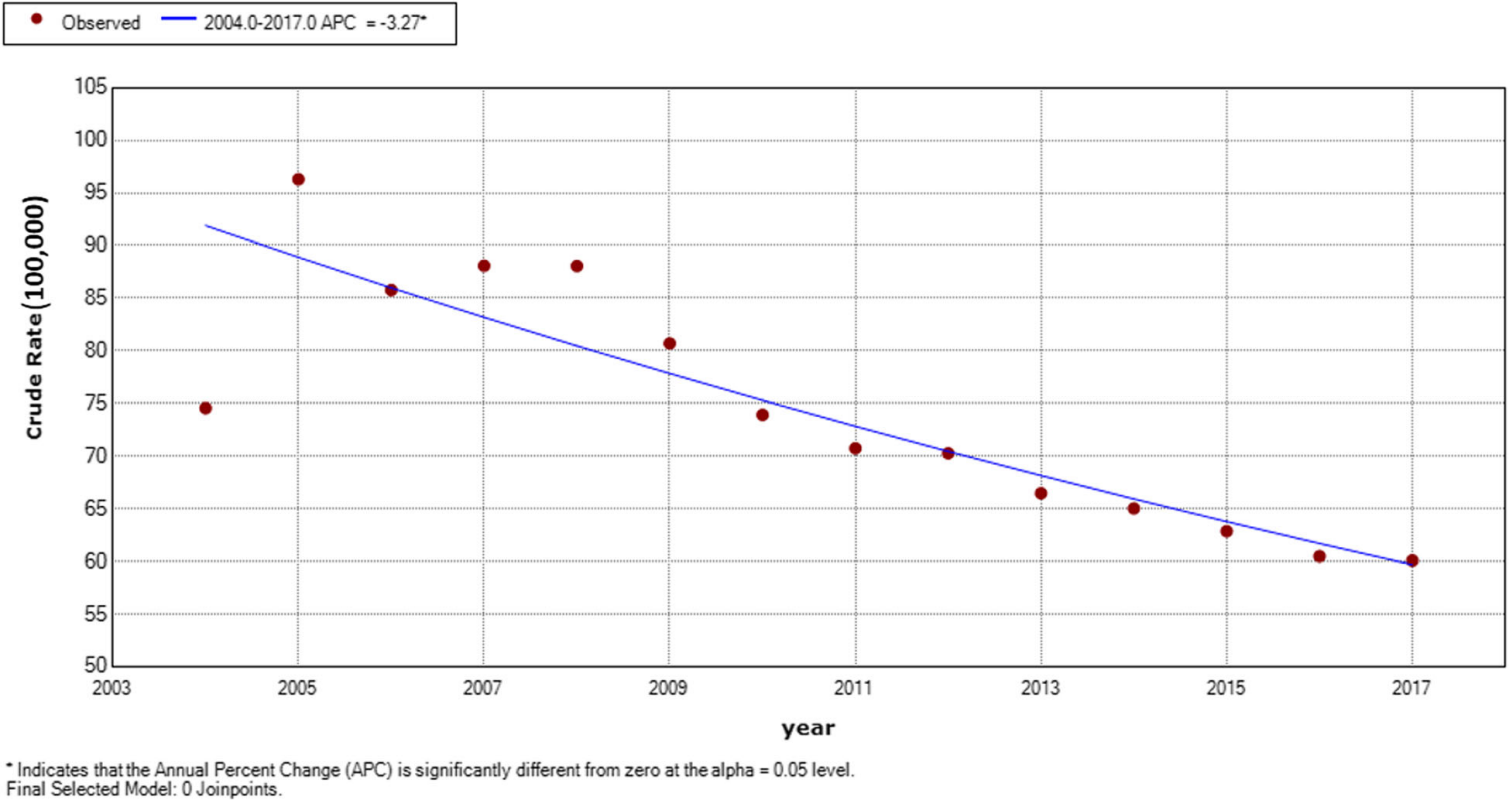
Trend of TB incidence rate from 2004 to 2017 shown by the Joinpoint software. The red squares denote the incidence of each year and the blue line is the slope of the annual percent change (APC)

We included 13,991,850 TB cases from January 2004 to December 2017, with a yearly average morbidity of 999,417 cases. A fluctuant reduction was seen from 74.57 (/100,000) cases in 2004 to 60.08 (100,000) cases in 2017, with the highest incidence of 96.30 (100,000) cases in 2005. The final model was the 0 Joinpoint model (*P* = 0.18). The annual average percent change (AAPC) was − 3.3 (95% CI: − 4.3 to − 2.2, *P* < 0.001) from 2004 to 2017, indicating a downward trend in the TB incidence (Fig. 1).

**Fig. 2:**
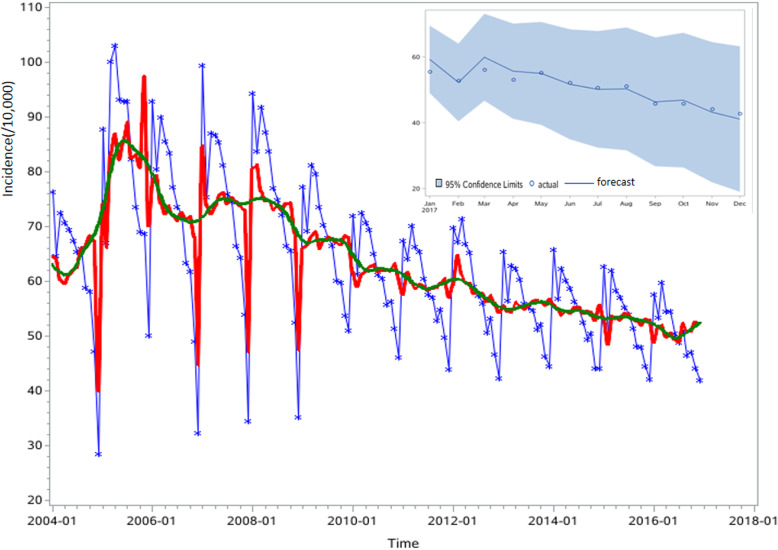
The actual and seasonal-adjusted incidence of TB in China, from January 2004 to December 2017 at monthly intervals. The blue line is the original incidence, the red line is the seasonal-adjusted incidence, and the green line is the trend line

The occurrence of TB with obvious seasonality was observed in the past 14 years (Fig. 2), and the seasonal cycle kept on fluctuating within 12 months. There were two incidence peaks in January and March every year, with a burst From December of the previous year to January of the following year.

**Fig. 3:**
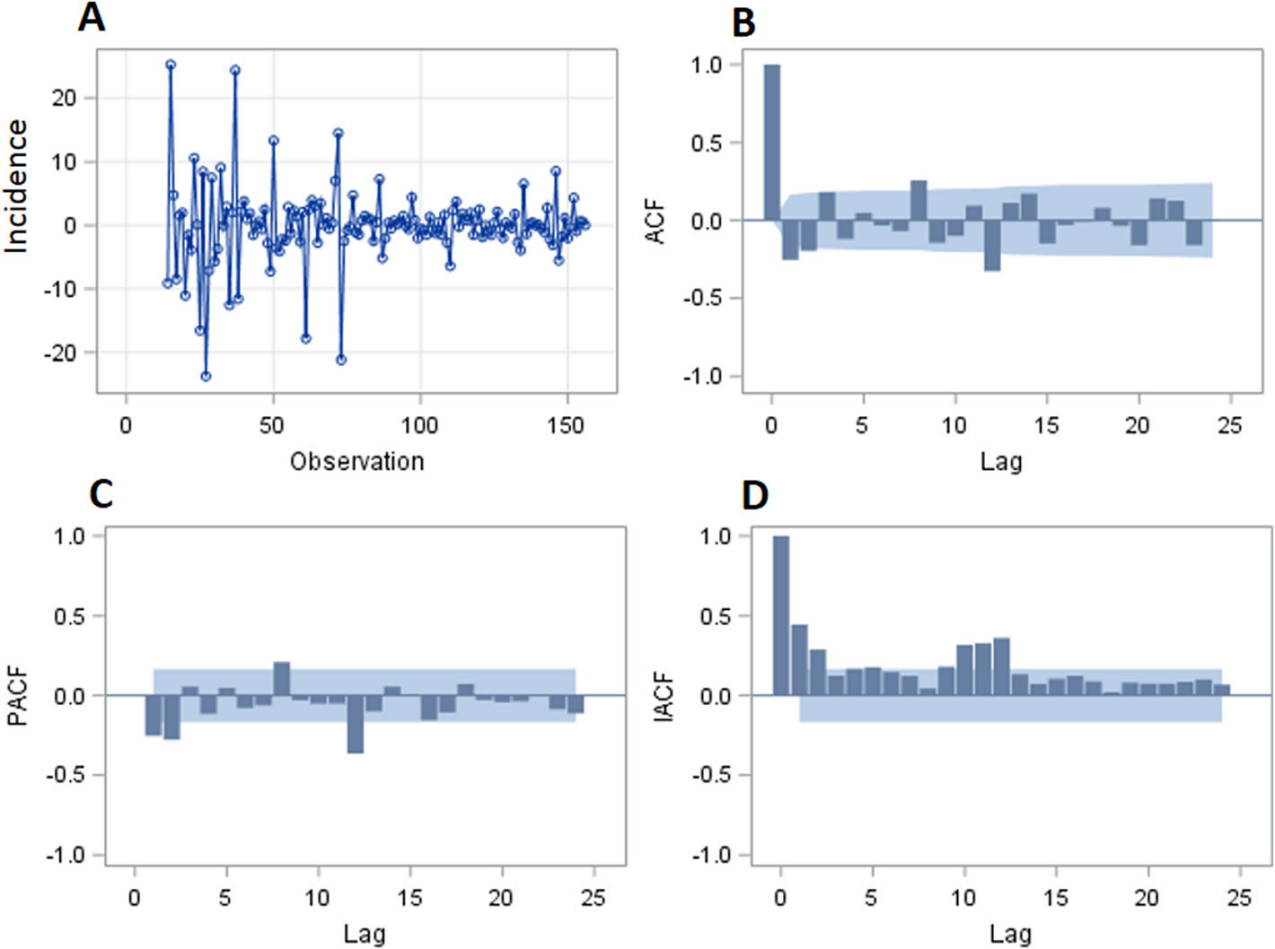
The time series of one step of 12 months difference and its three kinds of autocorrelation function plot. **a** The time series after one-step seasonal differences. The x-axis is the time and the y-axis is the difference between the value of incidence and the value a lag of 12 months. The plot (**b**–**d**) shows the degree of correlations with past values of the time series. For the plot (**b**–**d**), the x-axis is the number of periods of the lag, the y-axis is the coefficient of the autocorrelation, partial autocorrelation, and inverse autocorrelation, respectively. The blue shadows are the boundaries of confidence intervals (two times the standard deviation) of the coefficient. **b** The figure of the autocorrelation of the time series. **c** The figure of the partial autocorrelation of the time series. **d** The figure of the inverse autocorrelation of the time series

The null hypothesis of white noise was strongly rejected with the results of the white noise test (χ^2^ = 131.98, DF = 6, *P* < 0.0001), which can extract some useful information from the time series. Although the null hypothesis was significant (Tau = − 3.91, *P* = 0.003, lag = 1) for the augmented Dickey-Fuller (ADF) test, we should make a seasonal difference taking account of the fluctuation of the incidence figure. We performed a seasonal differencing to make sure that the transformed TB incidence was stationary (Tau = − 7.6, P < 0.0001, lag = 1) to better construct the SARIMA model (Fig. 3). Based on the figures of PACF, ACF, and IACF, the best ARIMA model was (0, 1, 1) X (0, 1, 1)_12_ which can be written as (1-B) (1-B^12^) X_t_ = (1–0.42349B) (1–0.43338B^12^) ε_t_, with a minimum AIC (880.5) and SBC (886.4). There was no significant correlation between residuals (lag = 6, χ^2^ = 3.65, DF = 3, *P* = 0.45), and the residual was a white noise. We then did an incidence forecast of 2017 shown in Fig. 2, the predicted and actual incidence were shown in Table 1. The predicted value and the original incidence data of 2017 were well matched. The mean square error (MSE), mean absolute percentage error (MAPE), root mean square error (RMSE), and mean absolute error (MAE) of the modelling performance were 201.76, 0.06, 14.2, and 8.4 respectively. The time series can divide into three components: seasonal effect, trend curve, and irregular noise. The seasonal effect refers to the fluctuations of the trend that is reproduced in a similar way every year, the trend curve is the long-term movement of the time series, and the irregular noise is the surplus component after trend curve and seasonal effect are removed. After eliminating the influence of seasonal effect and irregular noise on TB, the incidence curve of TB became smoother (Fig. 2), and it was found that the trend of the incidence from 2004 to 2016 was gradually decreasing.

**Table 1:** Actual and forecast data base on the SARIMA model of 2017 in China

Time	Actual data	Forecast	95% CI	
2017–01	55.55	59.2274	48.993	69.4617
2017–02	52.77431	52.2003	40.387	64.0136
2017–03	56.12181	59.8545	46.6497	73.0593
2017–04	53.10116	55.6036	41.1406	70.0667
2017–05	55.25359	54.9492	39.3289	70.5695
2017–06	52.24164	51.6317	34.9343	68.3292
2017–07	50.66754	50.1129	32.4037	67.8222
2017–08	51.08064	50.2320	31.5657	68.8983
2017–09	45.82495	46.2891	26.7124	65.8657
2017–10	45.77132	46.8417	26.3953	67.2882
2017–11	44.10445	43.1599	21.8792	64.4406
2017–12	42.79198	41.0710	18.9875	63.1545

### Spatial clustering distribution and geographic characteristics

**Fig. 4:**
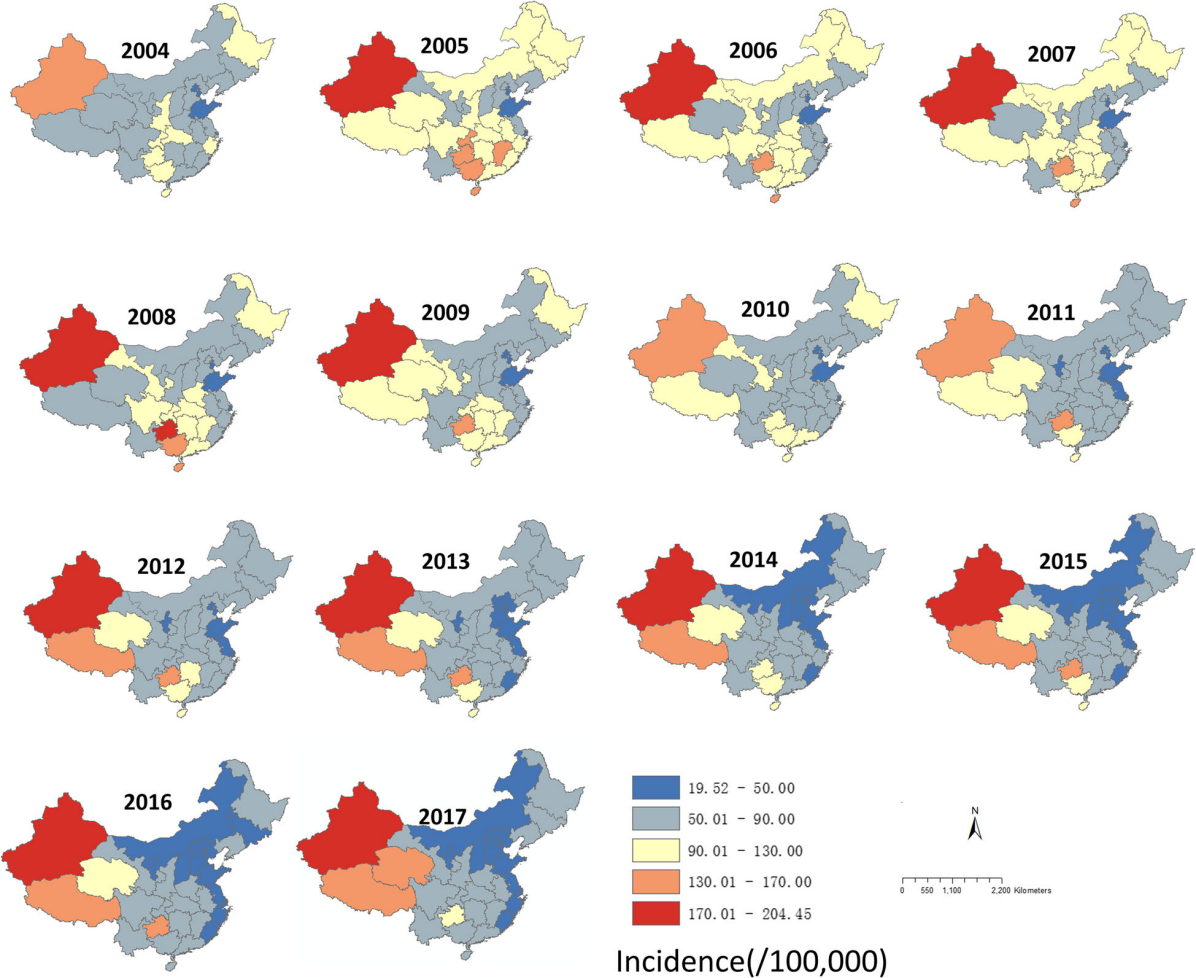
Maps of the incidence of TB in China, 2004–2017. Maps were created by ArcGIS software (version 10.1, ESRI Inc.; Redlands, CA, USA)

The TB cases were reported in every province of China from 2004 to 2017, with the lowest incidence of 19.52(/100,000) in Hebei Province (2015) to the highest incidence of 204.45(/100,000) in Xinjiang Province (2005). Xinjiang Province was the most prevalent province of Tuberculosis in China from 2004 to 2017, and the incidence of Tibet was in a high level since 2012 (Fig. 4). The provinces with a high incidence were located in the northwest (Xinjiang, Tibet) and south (Guangxi, Guizhou, Hainan) of China.

**Fig. 5:**
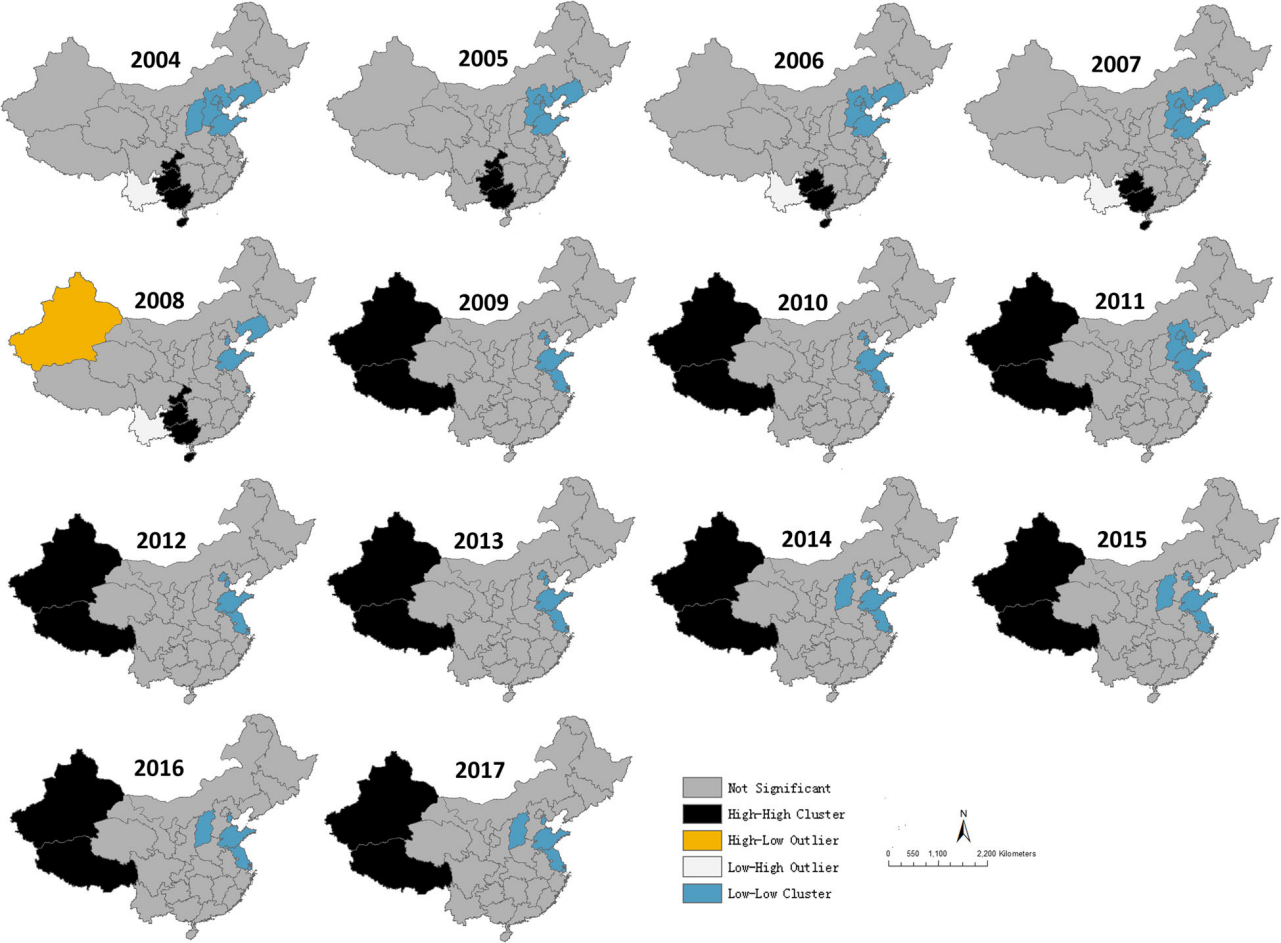
Maps of the local autocorrelation analysis of the incidence rate of TB in China, 2004–2017 by the local Moran’s I. Maps were created by ArcGIS software (version 10.1, ESRI Inc.; Redlands, CA, USA). The HH is the high-high spatial autocorrelation, the HL is the high-low spatial autocorrelation, the LH is the low-high spatial autocorrelation, and the LL is the low-low spatial autocorrelation

Based on the global autocorrelation analysis, the distribution of TB was spatially correlated from 2004 to 2017 (Table 2). The Moran’s index range from 0.28 to 0.36, and had the highest index in 2011(Moran’s index = 0.36, Z-score = 5.51, *P* < 0.001). According to the local Moran’s I autocorrelation results, it was found that there were totally 35 high-high clusters and 1 high-low cluster from 2004 to 2017 (Table 3), with 4, 3, 3, 3, 5, 2, 2, 2, 2, 2, 2, 2, 2, and 2 clusters each year. The hotspot of TB transmission was mainly located at southern region of China from 2004 to 2008, including Hainan, Guangxi, Guizhou, and Chongqing, which disappeared in the later years. It should be noted that the center of the high-high clusters moved from the East to the Northwest (Xinjiang and Tibet) after 2008, and Tibet was a high-low cluster in 2008 (Fig. 5).

**Table 2:** The global spatial autocorrelation of TB in China from 2004 to 2017

Year	Moran’s Index	Moran’s Z-score	*P*-value
2004	0.33	5.09	< 0.001
2005	0.35	5.45	< 0.001
2006	0.33	5.21	< 0.001
2007	0.34	5.36	< 0.001
2008	0.29	4.64	< 0.001
2009	0.31	4.99	< 0.001
2010	0.34	5.36	< 0.001
2011	0.36	5.51	< 0.001
2012	0.32	5.06	< 0.001
2013	0.32	5.11	< 0.001
2014	0.32	5.07	< 0.001
2015	0.30	4.79	< 0.001
2016	0.28	4.51	< 0.001
2017	0.28	4.54	< 0.001

**Table 3:** The local spatial autocorrelation of TB in China from 2004 to 2017

Year	Area	LMi Index	LMi Z-score	*P*-value	Correlation type	Incidence (/100,000)
2004	Guangxi	0.00001	2.82	0.005	High-High Cluster	119.62
2004	Hainan	0.000007	2.69	0.007	High-High Cluster	123.21
2004	Guizhou	0.00001	3.08	0.002	High-High Cluster	122.23
2004	Chongqing	0.000009	2.54	0.01	High-High Cluster	127.49
2005	Guangxi	0.00001	2.77	0.006	High-High Cluster	145.67
2005	Guizhou	0.00001	3.19	0.001	High-High Cluster	157.35
2005	Chongqing	0.000009	2.57	0.01	High-High Cluster	151.51
2006	Guangxi	0.000009	2.53	0.01	High-High Cluster	127.23
2006	Hainan	0.000006	2.40	0.02	High-High Cluster	140.54
2006	Guizhou	0.00001	2.86	0.004	High-High Cluster	146.21
2007	Guangxi	0.00001	2.72	0.007	High-High Cluster	129.84
2007	Hainan	0.000007	2.54	0.01	High-High Cluster	143.63
2007	Guizhou	0.00001	3.42	0.001	High-High Cluster	169.92
2008	Guangxi	0.00001	2.79	0.005	High-High Cluster	131.41
2008	Hainan	0.000006	2.47	0.01	High-High Cluster	139.16
2008	Guizhou	0.00001	3.47	0.0005	High-High Cluster	183.0
2008	Chongqing	0.000007	2.01	0.04	High-High Cluster	127.61
2008	Xinjiang	−0.000001	−2.02	0.04	High-Low Cluster	202.93
2009	Xinjiang	0.000002	2.40	0.0007	High-High Cluster	183.35
2009	Tibet	0.000002	2.28	0.02	High-High Cluster	118.3
2010	Xinjiang	0.000003	4.56	0.0001	High-High Cluster	164.46
2010	Tibet	0.000004	3.16	0.002	High-High Cluster	118.34
2011	Xinjiang	0.000003	4.87	0.00001	High-High Cluster	157.83
2011	Tibet	0.000004	3.79	0.0002	High-High Cluster	123.03
2012	Xinjiang	0.000004	6.18	< 0.00001	High-High Cluster	181.17
2012	Tibet	0.000006	5.45	< 0.00001	High-High Cluster	135.18
2013	Xinjiang	0.000004	6.61	< 0.00001	High-High Cluster	172.73
2013	Tibet	0.000006	5.79	< 0.00001	High-High Cluster	138.12
2014	Xinjiang	0.000005	7.54	< 0.00001	High-High Cluster	176.0
2014	Tibet	0.000007	6.32	< 0.00001	High-High Cluster	147.99
2015	Xinjiang	0.000004	6.66	< 0.00001	High-High Cluster	184.53
2015	Tibet	0.000007	6.56	< 0.00001	High-High Cluster	140.20
2016	Xinjiang	0.000005	7.95	< 0.00001	High-High Cluster	185.66
2016	Tibet	0.000009	8.14	< 0.00001	High-High Cluster	154.37
2017	Xinjiang	0.000005	8.41	< 0.00001	High-High Cluster	202.59
2017	Tibet	0.000009	8.43	< 0.00001	High-High Cluster	154.77

### Multivariate time series analysis

Two models following negative binomial distribution and the Poisson distribution constructed by the monthly data from 2004 to 2017were built in the first step, and the AIC of the two models were 72,247.32 and 260,511.37, which meant the better distribution of the model would be the negative binomial distribution. Second, we included the random effects of the model, and found that the random effects model (0.20) introduced by DSS rule was better than the negative binomial distribution model (2.06). Considering that most regions are very large, higher-order neighbourhood are not that relevant when we only construct the model with first-order neighbourhood.

**Fig. 6:**
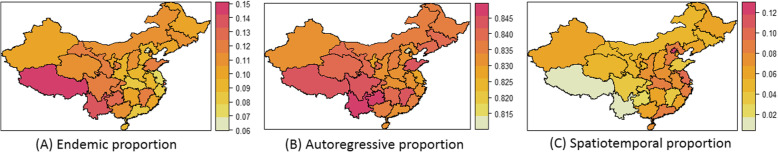
The three components of TB on average of fourteen years in the multivariate time series model. This map was created by R software (version 3.3.1, http://www.r-project.org/). The colors represented the value of the proportion of the three components at the province level

In order to classify the spatial-temporal effect of the TB, the relative importance of the model components by province, with an average of 14 years is shown in Fig. 6. The autoregressive component had a leading role in the incidence of TB which accounted for 81.5–84.5% of the patients across all provinces on average (Fig. 6b). The endemic component was about twice as large in the western provinces as the average while the spatial-temporal component was less important there (Fig. 6a/c). It should be noted that some economic circles, such as the Yangtze River Delta economic circle (Zhejiang, Jiangsu, and shanghai), Pearl River Delta economic circle (Guangxi and Guangdong), Bohai Economic Rim (Hebei, Tianjin, Beijing, and Shanxi) and Hanjiang ecological economic belt (Henan and Hubei), had higher proportions of the spatial-temporal component (especially in Beijing), whereas there was very little spatial correlation in the western provinces.

**Fig. 7:**
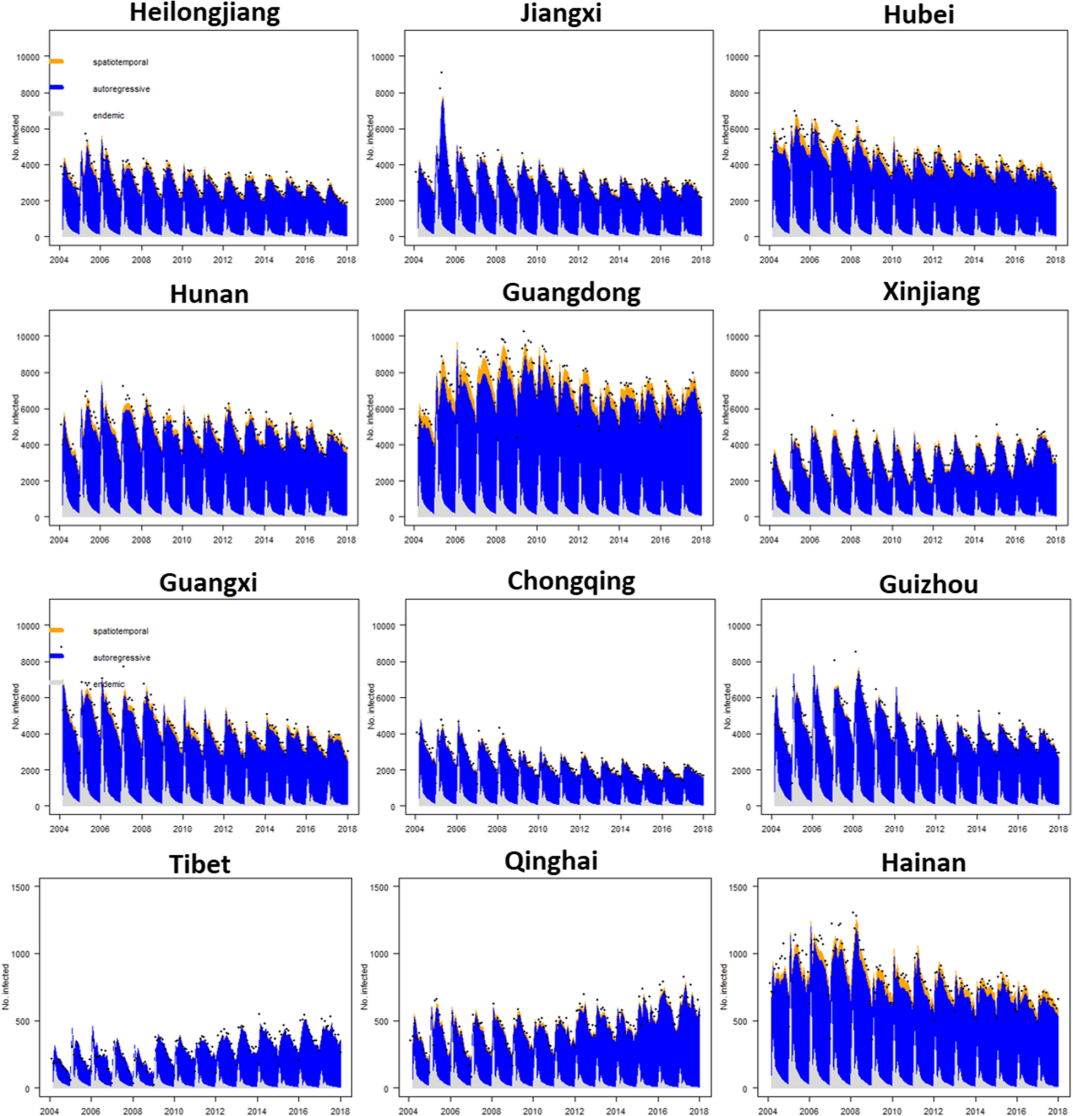
Fitted components in the multivariate time series model for the 12 counties with more than 70 cases during the past fourteen years. The black dots represent the monthly counts of incidence, the light grey area shows the endemic component, the blue area shows the autoregressive component, and the yellow area corresponds to the spatiotemporal component

An intuitive method to quantify the relative contributions of the high incidence regions (> 70 cases per 100,000 persons over 14 years) of the three components is provided by Fig. 7. In general, most of the high incidences were mainly affected by the autoregressive component for the past 14 years. There was clear seasonality with two incidence peaks in January and March every year, with a burst From December of the previous year to January of the following year. Guangxi, Heilongjiang, Hubei, Guangdong and Hainan were partly affected by the spatial-temporal component, while the rest of the high incidence provinces had nearly no associations with the spatial-temporal effect.

## Discussion

According to our research, there were 13,991,850 TB cases from January 2004 to December 2017, with a yearly average morbidity of 999,417 cases which was a huge burden for the public health of China. Understanding the epidemiology patterns of TB may help China to reduce the number of TB cases which ranked second in 2017 according to the WHO report [1]. The incidence of TB from 74.58 (/100,000) cases in 2004 to 60.08 (/100,000) cases in 2017 which was a 19.4% reduction of TB incidence. The annual average percent change (AAPC) was − 3.3, which is better than the world average of 2% [1]. The reason for the decline of TB incidence is the rising GDP (Gross Domestic Product) (China ranked second in 2019), high urbanization, and the widespread modern control strategy. Previous studies demonstrated that the TB incidence of China decreased with the rising of GDP and better healthy treatment and management [25, 26], which was also found in other countries [27, 28].

Consistent with previous research [9], we found two peaks in January and March every year for TB incidence in China, with close numbers in these two peaks. The low number of confirmed cases in February may probably attribute to the Chinese traditional Spring Festival holiday. The average time from disease onset to confirmation of the diagnosis was 72 days when some infected persons develop active Tuberculosis, and patients were most likely to be diagnosed 2–3 months after symptom onset [8]. So, we should enhance patient control and the prevention of susceptible population in the autumn and winter, and the detection of TB in spring.

For a long time, the hotspots were distributed in the northwest areas such as Xinjiang Province and Tibet. Xinjiang Province has been at a high incidence level in the 14 years, while the incidence in Tibet increased since 2012. Except for 2004, 2010, and 2014, Guizhou Province has been at a high incidence level in the later 11 years. Some provinces such as Hainan, Guangxi, and Chongqing were at a high incidence level before 2009, but have been at a low level since 2009. More attention is needed in these high incidence areas, especially in Xinjiang, Tibet and Guizhou, which may need more financial assistance. It should be noted that some High-High spatial autocorrelation including Hainan, Guizhou, Guangxi, and Chongqing Province have disappeared since 2009, while Xinjiang Province and Tibet have become new H-H regional areas since 2009. The possible explanation is the unbalanced economic development in these areas [29]. Some studies [30–32] have demonstrated that there has positive correlations between the poverty level of regions, families or individuals and the incidence of Tuberculosis.

At the average level of the province component over the 14 years, autoregressive components dominated all the provinces which can explain 81.5–84.5% of the incidence, while the spatiotemporal component was mainly located in the well-developed provinces. For some provinces such as Beijing, Jiangsu and other well-developed economic provinces which were partly affected by the spatiotemporal component, it is recommended to monitor TB infection of the floating population form the neighbouring areas. For example, individuals who work in Beijing but become infected with TB in their hometowns should stay at home before anti-Tuberculosis treatment and maintain the treatment for a couple of weeks, avoiding going to public places or having close contact with others. We also did an analysis for the provinces with a high incidence (> 70 cases per 100,000 over 14 years) of the three components. For the autoregressive component which dominated all the high incidence provinces, early protective implementation 2–3 months ahead of the peak could help us reduce the number of TB patients [8]. For the endemic parts, most infected patients could be explained by living conditions, ecological and climatological changes, and socioeconomic activities. Active treatment for TB patients and cutting off the pathway of transmission may be the most effective way to prevent TB [8, 33]. Another important method is increasing the public awareness, especially among old people and children, and enhancing their physical exercise, immunity, and general hygiene. In addition, the spatial-temporal component can also affect the transmission of TB. Guangxi and Guangdong Provinces, which are in the south-east coastal area, were partly influenced by the spatial-temporal component, indicating that these regions may have imported TB from adjacent country with high incidence such as Philippines [34, 35] or the neighbouring province Guizhou. Alarmingly, although there was no clear evidence that Tibet and Xinjiang had a high value of spatial-temporal component, we still need to pay attention to transmission from India [33, 34] which was ranked first in global TB patients.

Our study had several limitation. First, the monthly data from 2004 to 2017 did not collect some risk factors including socioeconomic status, climatic factors, gender, age, and human activities. The relationship between the incidence of TB and these factors was still unknown. These factors should be included in the future studies in order to get an accurate multivariate time series model. Second, we included TB patients reported from the passive surveillance system which inevitably underestimated the total number of TB cases. Further researches could consider the level of reporting, including some subclinical and mild individuals not accessing healthcare. Lastly, the level of diagnosis in some provinces can lead to an underestimation of the TB incidence. We should think over the diagnostic level in the future studies to correct the incidence.

## Conclusion

In conclusion, China still has a high TB incidence. However, the incidence rate of TB was significantly decreasing from 2004 to 2017 in China. Seasonal peaks were in January and March every year, with a burst From December of the previous year to January. Obvious geographical clusters were observed in Tibet and Xinjiang Province. The relative importance component of TB driving transmission was distinguished from the multivariate time series model. For every provinces over the past 14 years, the autoregressive component played a leading role in the incidence of TB which need us to enhance the early protective implementation.

## Data Availability

The original data can be found in the website of Chinese Center for Disease Control and Prevention (http://www.phsciencedata.cn/Share/edtShareNew.jsp?id=39208), and statistical yearbook of the National Bureau of Statistics (http://www.stats.gov.cn/tjsj/ndsj/).

## Abbreviations

TB: Tuberculosis
SARIMA: Autoregressive integrated moving average
AAPC: Annual average percent change
ACF: Autocorrelation function
PACF: Partial autocorrelation function
IACF: Inverse autocorrelation function
AIC: Akaike information criterion
SBC: Schwartz Bayesian Criterion
MSE: Mean square error
MAPE: Mean absolute percentage error
MAE: Mean absolute error.
RMSE: Root mean square error.
